# An Evening at the Meeting of the London Pathological Society

**Published:** 1868-08

**Authors:** 


### AN EVENING AT THE MEETING OF THE LONDON PATHOLOGICAL SOCIETY.

The London Pathological Society numbers among its membership many of the most valuable men in the medical profession in that city. Many in attendance upon its meetings are gray-haired and venerable, but the larger number are yet young men, who have risen by industry and talent to positions where they have abundant opportunities to pursue the study of pathology under the most favorable circumstances. Holding appointments as house-physicians and surgeons in the various hospitals, they are constantly coming upon cases whose histories, with verifications by autopsy, render them of intense interest to the profession. The aggregate of these labors furnishes abundant material to occupy the members at the stated meetings of the Society, and the only trouble is to find time to do justice to the cases presented. Men learn there the necessity of condensation in the statement of cases and the presentation of opinions; and the way the members manifest their desire to terminate a voluble harangue is so decided, that the bravest cannot long resist the pressure. A statement is always in the hands of the President, of just what is to be presented at that meeting; and the moment a case is called, the person having it in hand steps forward and claims attention. If brief, terse, and lucid, the more is he acceptable; if it has special merit, it receives most careful hearing; if trivial or trite, it is not wise for a young man to weary their patience long. So, too, the pet theories of old men are frequently cut short in the midst of their expression by the merciless *little bell.* If their presence is secured it is for a purpose, and there must be no abuse.

The effect is good every way. If a man has nothing worth saying, he soon learns to keep still; if his subject has merit, he strives to set it forth in brief, but enjoying, manner; and so, in a single evening, matters are brought under consideration and referred for publication in their Transactions, which a physician in private practice might only reach during long years of patient observation.

The regular sessions of the Society occur every two weeks. They are held in the rooms owned by the Medico-Chirurgical Society, from which they are rented for use upon these evenings. The London Obstetrical Society make use of the same rooms on other evenings in the same way. The rooms impress one as the theatre of action, where many of the best men that have lived, and most talented, have labored for the amelioration of misery; for the benefit of the race. The living are there in all the vigor of manhood; the memories of the dead are there also. The room in which the sessions are held is about 40 feet square, with high walls, covered on every side by cases of books. The lighting by day is accomplished entirely from the ceiling. On every side among the cases, on brackets, are placed marble busts, in memory of the venerable dead who once were members and officers of these societies. I made note of some of these, and of the years when they were presidents of the Medico-Chirurgical Society. I give a few of them as they were noted, but not in the order of their election. The likenesses are said, in most instances, to be very striking, and I could but think that our British brethren would do the world a favor if they would cause these busts to be photographed, that the profession in every land, who have admired these men while living, might now the better perpetuate their memories, by placing side by side their works and likenesses.

Among these, where all are chief, are the names of John Abernethy, President of the Society in 1823; of Benjamin Travers, in 1827; of William Laurence, in 1831; of Sir Astley Cooper, in 1819; of Sir Henry Halford, in 1810; of Gilbert Blain, in 1813; of William Babington, in 1817; of Thomas Addison, in 1849; of Sir Benj. Brodie, in 1839; of James M. Arnott, in 1847; of Sir Charles Locock, in 1857; of Cæsar H. Hawkins, in 1855; and other names, which space forbids me to write, to perpetuate the memory of whom receives the sanction of the world. But I must come to the meeting of the Society, which I attended on the 6th of May last.

The Society convened at 8 o'clock. Its President is John Simon, known to many Americans, and now holding the position as Chief of the Medical Department of the Privy Council, and having a general surveillance of epidemics, etc., as they occur in the kingdom, and giving direction as governmental attention is demanded in medical matters. I noticed that he attained the Astley Cooper prize in Guy's Hospital, in 1844, his subject being the "Thymus Gland." His preparations are carefully preserved in that museum, in accordance with the rules of the hospital. He makes a good presiding officer, and the utmost decorum was manifest. About 60 members were present, all very promptly in their seats, as though they came for a purpose and had business to transact. I give the minutes of some 20 of the 27 cases which had been enrolled in the order of their presentation. I am unable to give the names of the persons offering them.

*First, Second, and Third*, were specimens of morbid growths of the femur.

*Fourth.* Granular disease of the kidney. Capsule adherent; renal artery terminates in a cul-de-sac; weight 6 grains.

*Fifth.* Disease of the parietal bones, with specimens and history of the case. Skull thickened; brain covered with solidified lymph; patient sometimes violent in temper; no other disease manifest.

*Sixth.* Endocarditis. Specimen presented, case described; lymph shown on the tricuspid valves.

*Seventh.* Deformities of the chest—Pigeon-breast. Motion of heart discoverable through the thin walls at the fifth left costal interspace; alternates with recession of spaces on the right side.

*Eighth.* Perforation of chest—Pneumo-thorax.

*Ninth.* Four specimens of disease of the supra-renal capsule. The bronzing proportionate to the slowness, but not to the severity of the capsular disease.

*Tenth.* Multilocular ovarian tumor. Removed the previous day; description of its diagnosis and of operation; blood found in cysts; woman still living.

*Eleventh.* Tuberculated liver. Description of case; with remarks upon entozoa in the gall-bladder.

*Twelfth.* Follicular tumors upon parietal bone, with drawings. Semblance to malignant tumors; vascular; remarks on arrest of hemorrhage.

*Thirteenth.* Abdominal and thoracic aneurismal rupture, with description of sac. Difficulty of diagnosis in this case.

*Fourteenth.* Colloid cancer of stomach. Specimen presented; reasons for diagnosis. Referred to Cancer Committee.

*Fifteenth.* Ulceration of the larynx, with obstruction of the œsophagus.

*Sixteenth.* Epithelial cancer of the œsophagus and lung. Interest from deposit in the lung.

*Seventeenth.* Microscopic specimens, delineating inflammation of the mesentery of the frog. Stages of inflammation and the appearance of corpuscles; passage of white corpuscles through the veins; passage of a red corpuscle through thick vessel wall. Here occurred a lively discussion on the passage of corpuscles.

*Eighteenth.* Fracture of femur. Injury of vessels; gangrene following.

*Nineteenth.* Case of swallowing of date-stones, with strangulation of hernia, and passages of these from artificial opening in one month; gradual closure of intestinal opening.

*Twentieth.* Case of curious deposit in the lungs, liver, and kidneys.

*These*, with seven cases additional, were passed in rapid review, only those points which were unusual being seized upon and discussed briefly, as the cases suggested them. The meeting adjourned at *10 o'clock*, when a cold collation was served, according to custom, in the front parlor, or reception-room; the audience-room being on the first floor also, and in the rear of the former. An informal and spirited discussion was held among a few of the members after adjournment, having reference to the subjects presented with case *Seventeen.*

Prof. Lionel Beale, to whose courtesy and invitation I was indebted for the pleasure of attendance at the meeting, entered heartily into the discussion, as to the precise seat of the formation of pus corpuscles. His views, as given in his work on the growth of tissues, are being received with great favor, so far as I can judge, by those who have given them candid and careful study.

Such extended opportunities for study as these united efforts afford, are of untold value; and they account, in part, for the attainments of those men who have had to do very largely in shaping the medical literature and opinions of the world.

America, young in everything else, is yet in the infancy of such labors; but the facilities for improvement are more and more at hand, as our cities increase and our hospitals multiply. We may confidently anticipate that, as all eyes are now turned to a much more thorough and improved system of medical education, societies for medical improvement will also be multiplied, and that these, being wisely conducted, will enlist the best energies and talents of the profession, and secure for us an honorable position and a just respect by all who appreciate the value of medical attainment, both at home and in other lands.

The labors of the London Pathological Society are well worthy of emulation. It is in the power of our Chicago physicians and surgeons to institute a favorable comparison of efforts if they will. J. H. H.

---

HASTINGS, Minn., June 12, 1868.

PROFESSOR DAVIS,

*Dear Sir:*—At the meeting of the Morgan County Medical Society, in February last, in your State, the case of placenta prævia reported, led Dr. Prince to say that "he believed in this presentation, the profession would eventually adopt the maxim, 'the most speedy delivery possible.'"

With the highest respect for Dr. Prince, as a practitioner and a writer, I feel strongly impressed with the duty I owe to my fellow-beings, to give "mine opinion" and show a better way. Speedy delivery has two important objections: it is exceedingly dangerous, and in my opinion unnecessary. To many this will appear paradoxical; but it is, nevertheless, perfectly true. I am satisfied that a general resort to speedy delivery, as a main dependence, will result in the death of many women. My preceptor, in the State of New York, lost two cases, by that practice, out of three. My first was saved by that practice, but required very assiduous nursing and stimulating for many hours. Last fall we had two cases in this city, to both of which I was called in counsel. One was "delivered speedily" by another physician, of the most consummate skill, and proved fatal in half an hour. In the other case, we loosened the placenta and waited, and she delivered herself in three hours with entire safety—the hemorrhage ceasing upon the detachment of the placenta, and she gradually reviving from the state of depression to which she had been brought by the hemorrhage.

I think the article on this subject in Professor Simpson's Works, Vol. I., p. 599, one of the most complete pieces of good reasoning that I have ever seen; and that it should be in the hands of every practitioner of midwifery. I am an entire convert to his doctrine, and believe that his practice will save almost every case. An anxious study of this subject, and a practice of forty years, have led me firmly to this conclusion.

It would be quite improper to lengthen this paper, by attempting to give even a synopsis of Prof. Simpson's statements and arguments on this subject. I greatly hope that many of the readers of the EXAMINER will be led to possess themselves of a copy of his obstetrical works, and read it for themselves.

One of his positions is, that complete detachment of the placenta stops the hemorrhage which partial detachment produces. This looks inconsistent. To judge correctly of it, we must read his article and weigh his reasoning and facts.

He says the uterine vessels collapse as far as detachment takes place; and that the hemorrhage connected with partial detachment is through the placenta, and not directly from the uterine vessels. This I believe to be so.

To deliver a woman exhausted with hemorrhage, whilst we leave her still to bleed through a partially detached placenta, is one of the most dangerous operations that we can possibly subject her to. Is it not like amputating a thigh without a tourniquet? Or, after an accident, before reaction? It looks to me very much like these. Let me warn my brethren of the profession, therefore, how they proceed to "the most speedy possible delivery" in cases of placenta prævia, for it is often fraught with the most awful danger!

Save the blood of your patient with the tampon, cold, and rest, till you find delivery necessary. Then loosen the placenta, and wait for her to revive if depressed, and deliver herself if she can. If not, after reaction has taken place, so as to render it safe, deliver, and never before. The danger is from loss of blood. That, by detachment, is under our control. Immediate delivery can be but seldom necessary. It should not be resorted to to stop hemorrhage. If it be, it will often be followed by speedy death.

Mr. Editor, please excuse my plainness of speech, and assure my brethren of the continued esteem of an old man in the profession. Respectfully yours,

F. B. ETHERIDGE.

